# Telling the story of intersectional stigma in HIV‐associated Kaposi's sarcoma in western Kenya: a convergent mixed‐methods approach

**DOI:** 10.1002/jia2.25918

**Published:** 2022-07-12

**Authors:** Sigrid Collier, Rhea Singh, Aggrey Semeere, Helen Byakwaga, Miriam Laker‐Oketta, Devon E. McMahon, Linda Chemtai, Merridy Grant, Lisa Butler, Laura Bogart, Ingrid V. Bassett, Samson Kiprono, Toby Maurer, Jeffrey Martin, Naftali Busakhala, Esther E. Freeman

**Affiliations:** ^1^ Division of Dermatology University of Washington Seattle Washington USA; ^2^ Virginia Commonwealth University School of Medicine Richmond Virginia USA; ^3^ Massachusetts General Hospital Harvard Medical School Boston Massachusetts USA; ^4^ Infectious Disease Institute Makerere University Kampala Uganda; ^5^ Academic Model Providing Access to Healthcare Eldoret Kenya; ^6^ Centre for Rural Health University of KwaZulu‐Natal Durban South Africa; ^7^ Institute for Collaboration on Health Intervention and Policy University of Connecticut Storrs Connecticut USA; ^8^ RAND Corporation Santa Monica California USA; ^9^ Department of Internal Medicine, School of Medicine College of Health Sciences Moi University Eldoret Kenya; ^10^ Department of Dermatology Indiana University Indianapolis Indiana USA; ^11^ Department of Epidemiology and Biostatistics University of California San Francisco San Francisco California USA; ^12^ Department of Pharmacology and Toxicology, School of Medicine College of Health Sciences Moi University Eldoret Kenya

**Keywords:** stigma, Kaposi's sarcoma, HIV/AIDS, cancer, sub‐Saharan Africa, mixed methods

## Abstract

**Introduction:**

The experience of stigma can be multifaceted for people with HIV and cancer. Kaposi's sarcoma (KS), one of the most common HIV‐associated cancers in sub‐Saharan Africa, often presents with visible skin lesions that may put people at risk for stigmatization. In this way, HIV‐associated KS is unique, as people with KS can experience stigma associated with HIV, cancer, and skin disease simultaneously. The aim of this study is to characterize the intersectionality of HIV‐related, cancer‐related and skin disease‐related stigma in people living with HIV and KS.

**Methods:**

We used a convergent mixed‐methods approach nested within a longitudinal study of people with HIV‐associated KS in western Kenya. Between February 2019 and December 2020, we collected quantitative surveys among all participants and conducted semi‐structured interviews among a purposive sample of participants. Quantitative surveys were adapted from the abridged Berger HIV Stigma Scale to assess overall stigma, HIV‐related stigma, cancer‐related stigma, and skin disease‐related stigma. Qualitative data were coded using stigma constructs from the Health Stigma and Discrimination Framework.

**Results:**

In 88 semi‐structured interviews, stigma was a major barrier to KS diagnosis and treatment among people with HIV‐associated KS. Participant's stories of stigma were dominated by HIV‐related stigma, more than cancer‐related or skin disease‐related stigma. However, quantitative stigma scores among the 117 participants were similar for HIV‐related (Median: 28.00; IQR: 28.0, 34.0), cancer‐related (Median: 28.0; IQR: 28.0, 34.8), and skin disease‐related stigma (Median: 28.0; IQR: 27.0, 34.0). In semi‐structured interviews, cancer‐related and skin disease‐related stigma were more subtle contributors; cancer‐related stigma was linked to fatalism and skin‐related stigma was linked to visible disease. Participants reported resolution of skin lesions contributed to lessening stigma over time; there was a significant decline in quantitative scores of overall stigma in time since KS diagnosis (adjusted β = –0.15, *p* <0.001).

**Conclusions:**

This study highlights the role mixed‐method approaches can play in better understanding stigma in people living with both HIV and cancer. While HIV‐related stigma may dominate perceptions of stigma among people with KS in Kenya, intersectional experiences of stigma may be subtle, and quantitative evaluation alone may be insufficient to understand intersectional stigma in certain contexts.

## 1 INTRODUCTION

For people living with HIV and cancer, the experience of stigma is multifaceted, reflecting HIV and cancer‐related stigma simultaneously. Stigma is a well‐studied social construct, characterized by discrimination against individuals labelled as “other” because of socially undesirable characteristics [1]. Stigmatization of illnesses creates barriers to health equity and decreases quality of life among people with associated diseases [2]. Intersectionality was originally used to describe the marginalization of Black women who were targets of discrimination because of both race and gender identities [3]. The lens of intersectionality can also be used to understand the burden of multiple marginalized identities related to health states, such as HIV and cancer [3, 4]. Although HIV and cancer individually are known to be stigmatized health conditions associated with delayed healthcare‐seeking and poor treatment adherence [5, 6, 7, 8, 9], little is known about intersectional stigma among people with HIV, cancer, and Kaposi's sarcoma (KS) in resource‐limited settings [10, 11].

KS remains one of the most common HIV‐associated cancers, even in the era of antiretroviral therapy (ART) [12]. KS often presents with highly visible skin lesions, putting people at risk for stigmatization [13]. The intersectionality of HIV, cancer, and skin disease‐related stigma in people with HIV‐associated KS is of particular interest, as all three conditions are known to be associated with stigma [10, 13]. The manifestations of these three intersecting stigmas have the potential to impact healthcare engagement at many levels, including delayed diagnosis and treatment of KS.

The goal of this study was to describe the prevalence and experiences of stigma in a longitudinal cohort of adults with newly diagnosed HIV‐associated KS in western Kenya using a mixed‐methods approach, guided by the Health Stigma and Discrimination Framework [14]. We aimed to characterize: (1) manifestations of stigma in HIV‐associated KS; (2) intersectionality of HIV‐related, cancer‐related, and skin disease‐related stigma in KS; and (3) longitudinal changes in the experience of stigma in HIV‐associated KS following diagnosis.

## 2 METHODS

### 2.1 Study population

This study was nested within a parent epidemiologic study using the rapid case ascertainment approach to identify and enrol all people newly diagnosed adults (aged 18 years or older) with KS from 2016 to 2019 at the Academic Model Providing Access to Healthcare (AMPATH) network in western Kenya [15]. KS was diagnosed histopathologically; a clinical diagnosis was made when biopsy was deemed unsafe (e.g. oral/ocular KS). Participants were followed longitudinally at 16‐week intervals to assess survival and other measures, including stigma. These time intervals were selected to match clinical care follow‐up visits in oncology. The study was approved by the Institutional Research Ethics Committee at Moi University and Partners Healthcare Institutional Review Board; all participants provided written informed consent.

### 2.2 Data collection procedures

#### 2.2.1 Physical examination, questionnaires and blood draws

Eligible participants completed questionnaires on demographics and KS‐related symptoms, underwent physical examination (total body skin examination, lymph node palpation, assessment of lymphedema, and pulmonary, abdominal and cardiac examination when indicated), and provided biological samples to assist in KS staging and categorization of co‐variates [15].

#### 2.2.2 Quantitative, abridged Berger HIV Stigma Scale

To measure distinct forms of stigma experienced by people with HIV‐associated KS, we adapted the validated abridged 25‐item Berger HIV Stigma Scale (Berger‐aHSS) to create four quantitative surveys evaluating (1) overall stigma, (2) cancer‐related stigma, (3) skin disease‐related stigma, and (4) HIV‐related stigma [16, 17] (Supplement A). The Berger HIV Stigma Scale (HSS) was selected because the original Berger HSS has been adapted and validated for use around the world [16, 18], in sub‐Saharan Africa [19, 20], and has been successfully used to study non‐HIV health conditions, including cancer [21, 22]. The adapted quantitative surveys were translated to Swahili, back‐translated to English and finally, field tested in western Kenya by research staff (Supplement A).

The Berger‐aHSS includes sub‐scores of the following four constructs from the Health Stigma and Discrimination Framework [14]: internalized stigma (6‐items, range 6–24), experienced stigma (10‐items, range 10–40), perceived stigma (4‐items, range 4–16), and anticipated stigma (5‐items, range 5–20) [16, 17]. Each item on the Berger‐aHSS is a 4‐point Likert scale (1‐strongly disagree, 2‐disagree, 3‐agree and 4‐strongly agree), with a range of composite scores for the entire quantitative survey between 25 and 100; higher scores indicated higher stigma.

Overall stigma was measured at enrolment. At follow‐up visits every 16 weeks, participants completed the quantitative surveys (*overall stigma*, *cancer‐related stigma*, *skin disease‐related stigma*, and *HIV‐related stigma*). All living participants in the parent study were asked to complete the adapted Berger‐aHSS scales from February 2019 to December 2020. Follow‐up study visits to collect the adapted Berger‐aHSS scales occurred at 16‐week intervals for the first 2.5 years after KS diagnosis.

#### 2.2.3 Qualitative, semi‐structured interviews

From February 2019 to August 2019, a purposive sample of participants with newly diagnosed KS from the parent study were invited to participate in in‐depth interviews focused on barriers and facilitators to diagnosis and treatment of KS (Supplement B). The interviews included probes on the role of stigma as a barrier to KS diagnosis, initiation of and adherence to chemotherapy, and the intersection of HIV‐related, cancer‐related, and skin disease‐related stigma in people with KS (Supplement B). Research staff trained in qualitative interview techniques conducted in‐person, in‐depth, semi‐structured interviews. Participants were compensated for transportation to the clinic. All interviews were approximately an hour and audio recorded.

### 2.3 Analysis

#### 2.3.1 Quantitative analysis

We used descriptive statistics to summarize baseline demographic and clinical characteristics. For each of the adapted Berger‐aHSS quantitative surveys, we calculated a total additive composite score and categorized total scores based on percentile of total possible composite scores. “Mild” was between 25th percentile and 50th percentile (25–50), where the average response was “strongly disagree” or “disagree,” “Moderate” was between 50th and 75th percentile (51–75), where the average response was on the border between “disagree” and “agree,” and “Severe” values were greater than 75th percentile (76–100), where the average response was “agree” or “strongly agree” to the questions about stigma in the Berger‐aHSS [23]. We used the same percentile criteria to categorize sub‐scores for each of the domains in the Berger‐aHSS as “Mild,” “Moderate” and “Severe.”

We performed exploratory analyses focused on understanding missingness in the data, differences in stigma over time and the relationships between overall, cancer‐related, HIV‐related, and skin disease‐related stigma. To evaluate longitudinal changes in overall stigma in people with HIV‐associated KS after diagnosis, we used a linear mixed‐effects model with a Gaussian link, where the intercept for each participant was assessed as a random effect, and age, sex, KS stage at the time of diagnosis; death at the end of the study period, and baseline CD4^+^ T cell count were included as fixed‐effects. Time was parameterized as the time from KS diagnosis (either biopsy date or date of clinical diagnosis, if biopsy was not performed) to the date when the adapted Berger‐aHSS quantitative surveys were completed. All analyses were performed using R statistical analysis software [24].

#### 2.3.2 Qualitative analysis

The recorded semi‐structured interviews were transcribed in the language in which the interview was performed (Swahili, English or local dialect) by trained Kenyan research assistants, and translated into English when necessary. Framework analysis was implemented using the Health Stigma and Discrimination Framework, focused on the Stigma Experiences constructs [14, 25, 26]. A priori coding framework was developed, and the first 25% of transcripts were coded independently by two experienced qualitative analysts (DM and MG) of the research team, using both inductive and deductive methods to verify and ensure reliability of the coding process. Coding structures were iteratively compared, with any discrepancies resolved by consensus. A single coder then coded the remaining 66 transcripts using the master codebook, with additional generation of codes, memos and interview summaries. A total of 88 interview transcripts; 31 diagnosis and 57 treatment, were analysed. NVivo (Version 12) was used to facilitate analysis. Codes were then grouped into themes with respective quotes.

Each component of KS (HIV, cancer, and skin disease) was analysed for five different stigma constructs as follows: (1) anticipated: the anticipated fear of what would happen if others knew about the person's disease, (2) perceived: the stigma associated with each person's understanding of how others in their community feel about them and their disease, (3) experienced: the person's experience of discriminatory acts or behaviors, (4) internalized: the person absorbed and applied to themselves the negative messages or stereotypes about their illness and (5) secondary stigma: the stigma experienced by those close to the person.

#### 2.3.3 Mixed methods; integration of qualitative and quantitative

We triangulated the quantitative and qualitative results to understand convergence and divergence and developed joint displays. Secondary stigma was not captured by the Berger‐aHSS nor the qualitative interviews and was therefore not included in the final analysis.

## 3 RESULTS

### 3.1 Quantitative

We enrolled 182 adults with newly diagnosed HIV‐associated KS at AMPATH. A total of 64.3% (*N* = 117/182) of participants completed the adapted Berger‐aHSS measuring *overall stigma* during at least one study visit. The median age of participants who completed at least one adapted Berger‐aHSS measuring *overall stigma* was 37 years (IQR 31, 42), 67% (*N* = 78) were men (Table 1). Details of participant characteristics and loss to follow‐up are included in Supplement A.

**Table 1:** Characteristics of participants living with HIV‐associated KS diagnosed at AMPATH in western Kenya 2016–2019, with at least one stigma measurement during the study period

	Mean (SD), Median (Q1, Q3) or *N* (Percentage)	
Characteristic	Participants with stigma measure (*N* = 117)	Participant without stigma measure (*N* = 97)
Age	37.0 (31.0, 42.0)	36.0 (32.0, 43.3)
Male sex	78 (67.2%)	59 (61.5%)
CD4^+^ T cells, cells/μl at diagnosis	342.9 (264.0)	283.5 (290.7)
ACTG stage at diagnosis		
T1	102 (87.2%)	89 (92.7%)
T0	15 (12.8%)	7 (7.3%)

Abbreviations: ACTG, AIDS Clinical Trials Group; SD, standard deviation; Q1, quartile 1; Q3, quartile 3. “T” denotes ACTG tumor stage.

**Figure 1:**
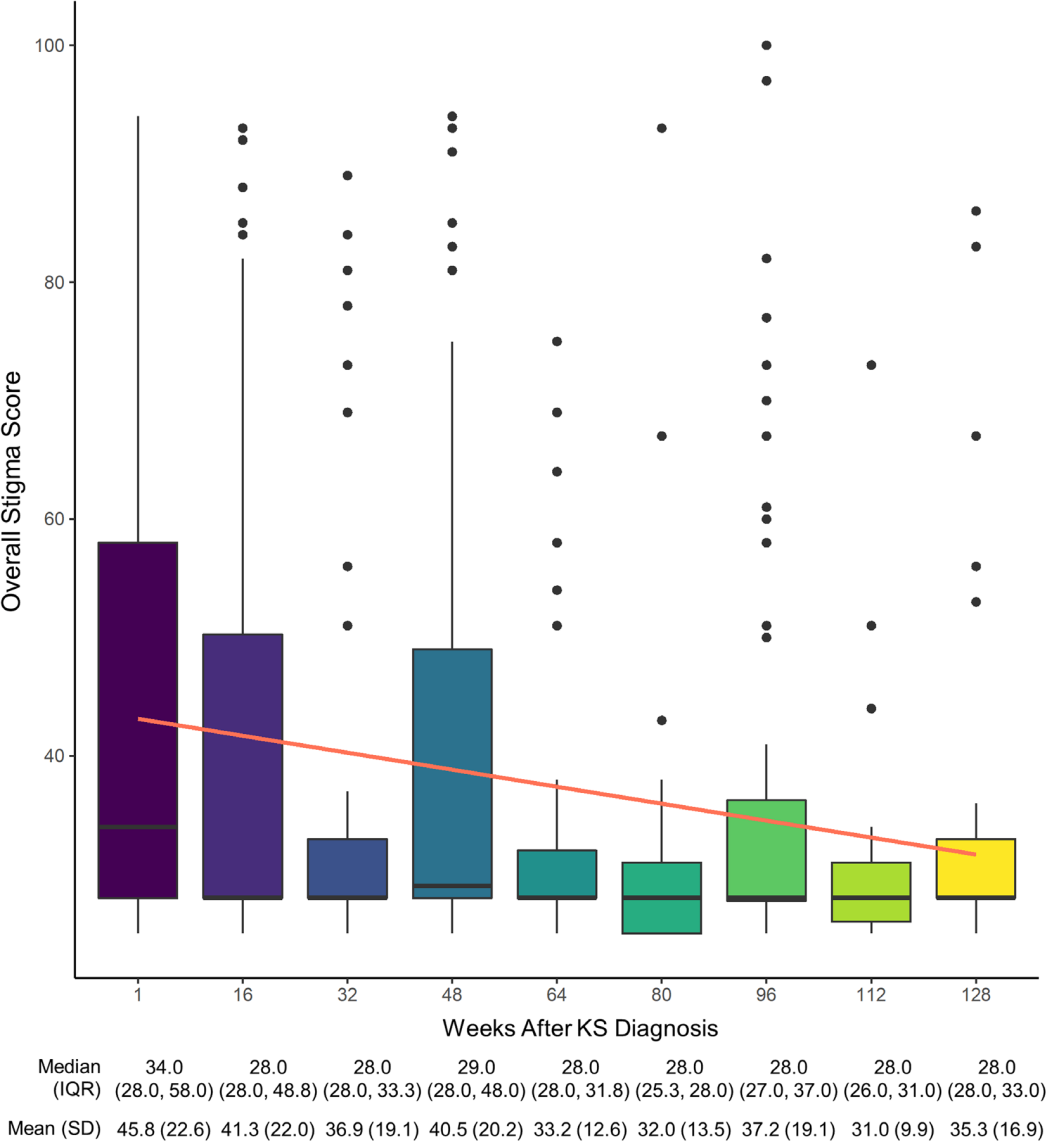
Longitudinal trend in overall stigma score among people with HIV‐associated Kaposi's sarcoma. Boxplot and linear regression depicting overall stigma scores by week. Note: The boxes represent the interquartile range (25th and 75th percentiles), and the median is depicted as a black horizontal line. The whiskers show the 0.35th and 99.65th percentiles. The outliers are depicted as black dots. The linear regression and 95% confidence intervals are depicted as the orange line and gray shadow, respectively. Abbreviations: IQR, interquartile range; KS, Kaposi's sarcoma; SD, standard deviation.

The median overall stigma score across all participants at all nine timepoints was 28.0 (IQR 28.0, 38.0). For overall stigma, the median sub‐scores for the constructs measured by the Berger‐aHSS were as follows (Table 2): internalized stigma was 6.00 (IQR 6.00, 9.00), perceived stigma was 4.00 (IQR 4.00, 7.00), anticipated stigma was 8.00 (IQR 7.00, 10.0), and experienced stigma was 10.0 (IQR 10.0, 13.0) (Figure 1) [16, 17].

**Table 2:** Stigma and intersectional stigma across all time points in participants with HIV‐associated Kaposi's sarcoma, as measured by adaptations of the Berger‐aHSS scale

	Overall stigma	Cancer stigma	HIV stigma	Skin disease stigma
	(*N* = 421)	(*N* = 368)	(*N* = 368)	(*N* = 368)
Stigma category				
Mild	336 (79.8%)	302 (82.1%)	313 (85.1%)	307 (83.4%)
Moderate	48 (11.4%)	29 (7.9%)	25 (6.8%)	24 (6.5%)
Severe	37 (8.8%)	37 (10.1%)	30 (8.2%)	37 (10.1%)
Overall score				
Mean (SD)	38.08 (19.07)	37.26 (19.37)	36.25 (17.97)	36.60 (19.04)
Median (Q1, Q3)	28.00 (28.00, 38.00)	28.00 (28.00, 36.00)	28.00 (28.00, 34.00)	28.00 (28.00, 34.00)
Self‐stigma				
Mean (SD)	8.51 (4.61)	8.36 (4.68)	7.98 (4.36)	8.18 (4.57)
Median (Q1, Q3)	6.00 (6.00, 9.00)	6.00 (6.00, 8.00)	6.00 (6.00, 7.00)	6.00 (6.00, 7.25)
Perceived stigma				
Mean (SD)	6.11 (3.57)	5.99 (3.63)	5.57 (3.25)	5.82 (3.53)
Median (Q1, Q3)	4.00 (4.00, 7.00)	4.00 (4.00, 7.00)	4.00 (4.00, 5.00)	4.00 (4.00, 6.00)
Anticipated stigma				
Mean (SD)	9.09 (3.83)	8.74 (3.88)	9.39 (4.10)	8.71 (3.83)
Median (Q1, Q3)	8.00 (7.00, 10.00)	8.00 (5.00, 8.00)	8.00 (8.00, 12.00)	8.00 (5.00, 8.00)
Experienced stigma				
Mean (SD)	14.37 (8.61)	15.56 (9.51)	14.69 (8.83)	15.27 (9.40)
Median (Q1, Q3)	10.00 (10.00, 13.00)	11.00 (11.00, 13.00)	11.00 (11.00, 11.00)	11.00 (11.00, 11.00)

Abbreviations: SD, standard deviation; Q1, quartile 1; Q3, quartile 3.

Median stigma scores were similar for the three stigmas experienced by people with HIV‐associated KS: HIV‐related stigma was 28.00 (28.0, 34.0), cancer‐related stigma was 28.0 (28.0, 34.8), and skin disease‐related stigma was 28.0 (27.0, 34.0). The median overall stigma score was highest at baseline with a median of 34.0 (IQR 28.0, 58.0) and lowest at week 112 with a median of 28.0 (IQR 26.0, 31.0) (Figure 1). There was a statistically significant longitudinal decrease in overall stigma following KS diagnosis. Specifically, overall stigma score decreased by –0.15 +/– 0.028 points for each additional week following KS diagnosis after accounting for random intercepts by participant and fixed‐affects for sex, age, KS stage at the time of diagnosis, death at the end of the study period and baseline CD4 count (95% CI: –0.21, –0.098; *p* < 0.001) (Supplement A, Table S1). Detailed regression analysis, including sensitivity analysis, is included in Supplement A.

### 3.2 Qualitative results

**Figure 2:**
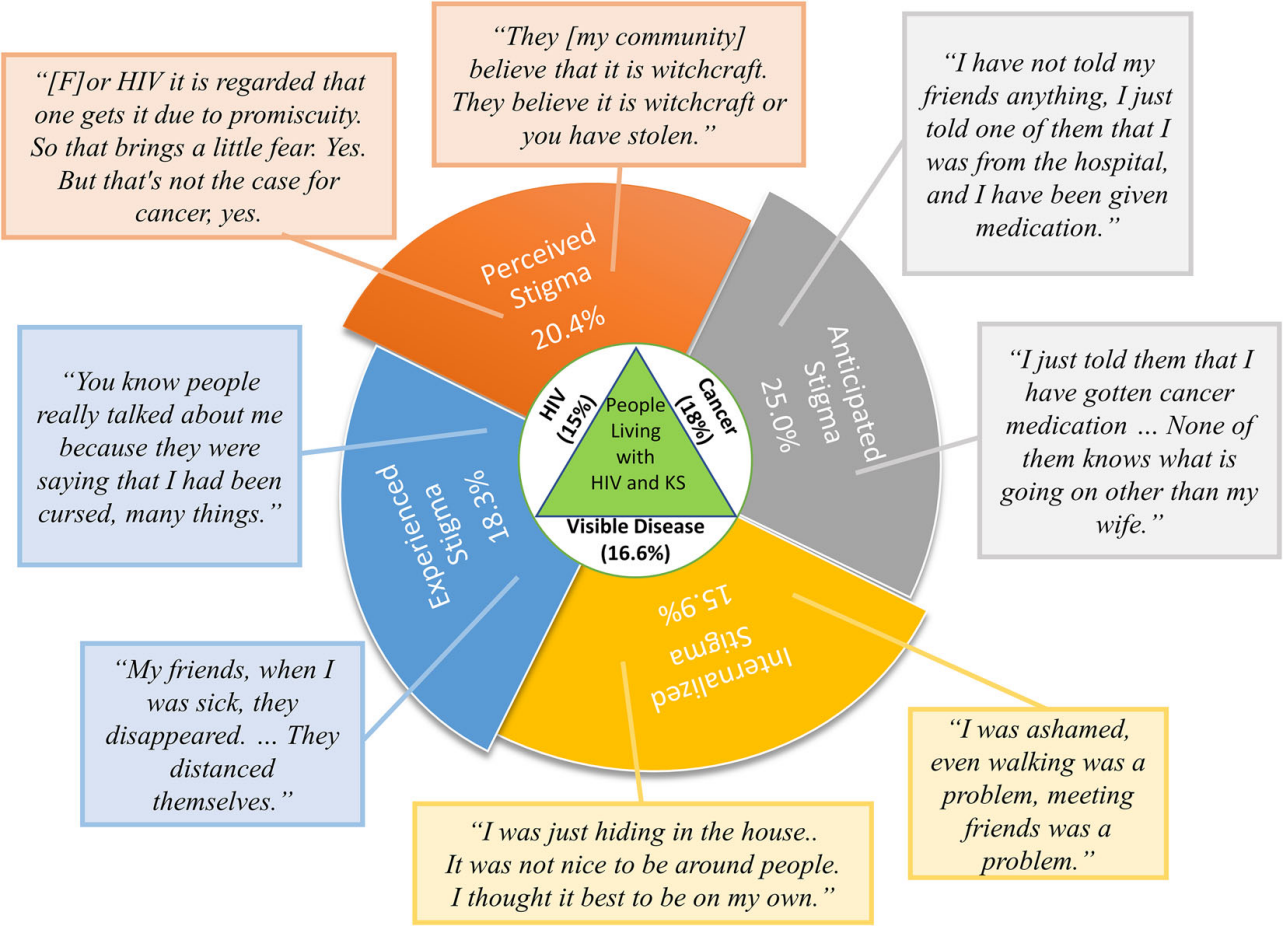
Mixed‐methods representation of stigma in HIV‐associated Kaposi's sarcoma: manifestations and proportion experiencing moderate or severe overall stigma. Joint display of the manifestations of stigma described by the Health Stigma and Discrimination Framework, with each portion of the pinwheel representing one of the stigma constructs (perceived, anticipated, internalized and experiences) included in the analysis. The quantitative results are represented as the percentage of responses with moderate or severe stigma for each of the constructs. The call outs extending from each of the pinwheels include representative quotes for each construct.

Stigma was an important aspect of people with KS's lived experience, and among people with HIV‐associated KS, the manifestations and degree of stigma varied among the three co‐occurring stigmatized diseases (HIV, cancer and skin disease) (Figure 2). HIV‐related stigma, specifically anticipated stigma around HIV‐disclosure, was the most common type of stigma and the most concerning stigma among many participants. Cancer‐related stigma was less commonly spontaneously expressed by participants; however, there were many stories of experienced cancer‐related stigma, manifesting as social isolation and discrimination related to their cancer diagnosis.

Skin disease‐related stigma was also a common theme, characterized by experienced stigma, which manifested as people staring and distancing themselves, and internalized stigma, manifesting with embarrassment and shame due to KS‐related skin changes, drainage and odor.

### 3.3 HIV stigma

In semi‐structured interviews, HIV‐related stigma was the most common form of stigma expressed by people with KS. Many participants mentioned perceiving high levels of HIV stigma in the community. They were fearful of disclosing their HIV status to friends and family (anticipated stigma) and some recounted being left by their romantic partner after disclosing their status (experienced stigma).

#### 3.3.1 Perceived HIV stigma

People with KS were more concerned with the public perception of HIV than of cancer and even skin disease. In particular, participants identified HIV as having a “bad name” and being associated with perceived “promiscuous” behaviour. *“I was not afraid of cancer but HIV, it is still a strong name. Even now people see cancer as a normal thing, but HIV is a bit different…People take cancer as a normal thing… .”* (Participant 53, 34‐year‐old man, new HIV diagnosis) *“[F]or HIV it is regarded that one gets it due to promiscuity. So that brings a little fear. Yes. But that's not the case for cancer, yes. (Laughs)”* (Participant 76, 39‐year‐old woman, new HIV diagnosis)

#### 3.3.2 Anticipated HIV stigma

Participants expressed fear about HIV status disclosure, anticipating that friends would distance themselves and family members might leave because of their HIV status. *“Up to now I don't want them to know that I have… that I tested positive. I don't want them to know, but I don't mind them getting to know about cancer.”* (Participant 76, 39‐year‐old woman, new HIV diagnosis).*“[I] was afraid of telling my wife because of—I tested positive [for HIV].”* (Participant 31, 28‐year‐old man, previous HIV diagnosis)

#### 3.3.3 Experienced HIV stigma

People with KS expressed fewer direct experiences of HIV‐related stigma than for cancer‐related and skin disease‐related stigma, especially when their status was undisclosed. However, among participants who disclosed their status, friends and family would often distance themselves or leave:

*[W]henever I went to take my medication, my wife would throw them away, so it got to a point where I got tired and decided to just quit taking them. When she saw the state that I was in she left, she left the children and went away*. (Participant 25, 29‐year‐old man, previous HIV diagnosis)

While rare, some individuals reported stigma from the healthcare workers involved in their care. One participant, who was pregnant, reported poor treatment from healthcare workers when her inability to take anti‐retroviral medications caused an increase in her viral load:

*…I was expecting [a child], that is what brought all the problem, the viral load started going up, taking the drugs was also a challenge, when I just tried to take them [ARVs] I could vomit so I wasn't taking them as required*. (Participant 19, 39‐year‐old woman, previous HIV diagnosis)

When asked if the doctors spoke to her poorly, she responded *“Yes, they talked to me so badly, I had a really heavy heart, I tried removing it [the pregnancy], but I could not.”* (Participant 19, 39‐year‐old woman, previous HIV diagnosis)

#### 3.3.4 Internalized HIV stigma

Fewer participants expressed internalized HIV‐related stigma explicitly, though HIV diagnosis was associated with feelings of despair, devaluation of their life, and on occasion, suicidal thoughts.

*For example, one patient said, “I was really scared because I thought…Where did I get it? How … could I have gotten it?’ It really disturbed me I almost hanged myself.” Subsequently, when asked if this fear made her lose hope about starting treatment, she acknowledged she had lost hope, “Yes, I thought it was best if I died.”* (Participant 84, 30‐year‐old woman, previous HIV diagnosis)

### 3.4 Cancer stigma

The manifestations of cancer‐related stigma among people with KS were subtle and intertwined with a sense of fatalism and fear of death associated with their cancer diagnosis. Although most participants expressed HIV‐related stigma overshadowing any cancer‐related stigma, many participants experienced discrimination they associated with their cancer diagnosis.

#### 3.4.1 Perceived cancer stigma

There were mixed perceptions of cancer stigma in the community. Although there was a stigmatizing fear among some community members that cancer was contagious, some participants felt that because KS is a cancer specifically linked to HIV status, this precluded them from additional cancer‐related stigma:

*I was not stigmatized by the cancer because the Google stated to me clearly that this is a cancer related to HIV so it's not just a normal cancer I would have been stigmatized if this is a cancer which is not related with HIV, that is why I was very comfortable, this is a cancer related to HIV, then its fine, because I am HIV*. (Participant 23, 43‐year‐old man, previous HIV diagnosis)

#### 3.4.2 Anticipated cancer stigma

In contrast to anticipated HIV‐related stigma, where fear of HIV status disclosure was common, no participants explicitly stated the notion of anticipated stigma related to their cancer diagnosis. *“When they told me that I had cancer I was open to our family. Even now everyone knows that I had cancer.”* (Participant 58, 36‐year‐old woman, previous HIV diagnosis)

#### 3.4.3 Experienced cancer stigma

Participants only rarely described experienced stigma associated with their cancer diagnosis. However, many people noted that family and friends distanced themselves after learning about the cancer diagnosis, because of the need for money and other help with cancer treatment and beliefs that death from cancer was inevitable. *“[…] people kept away from me and like I said, the reason was because of the money needed for my treatment was a lot. […] not because I had cancer.”* (Participant 54, 40‐year‐old man, new HIV diagnosis)

The relationship between cancer and death was a common theme and subtle component of the experiences of discrimination and distancing by family and friends. *“Cancer, you know cancer kills.”* (Participant 76, 39‐year‐old woman, new HIV diagnosis)

#### 3.4.4 Internalized cancer stigma

Internalized cancer‐related stigma was expressed by feelings of uselessness and hopelessness. Many participants expressed feeling their life was no longer worth living, and cancer was a death sentence. Participants also expressed feeling useless after their cancer diagnosis as their health worsened and they became dependent on others for financial and psychosocial support. *“Of course, I understood that my life is just useless, that means I am here for nothing, I cannot support my family, I cannot support myself, I saw that I was becoming useless.”* (Participant 59, 44‐year‐old man, new HIV diagnosis)

### 3.5 Skin‐disease stigma

Skin changes are often the most prominent visible manifestations of KS, and stigma was more commonly associated with certain skin changes, such as swelling, weeping and odor than with the purple patches and skin nodules. Similar to the experience of cancer‐related stigma, most participants did not express concerns about negative perceptions of skin disease by community members, but participants did hide their skin disease if possible (anticipated stigma), expressed embarrassment and shame because of skin changes (internalized stigma), and experienced staring and other subtle forms of stigmatization (experienced stigma).

#### 3.5.1 Perceived skin‐disease stigma

Similar to cancer‐related stigma, most participants did not express concerns about community members having negative perceptions of their skin disease, though some communicated fear over how others would react to their skin lesions. One person reported he worried about *“how they [others] would react”* to the “*spots.”* (Participant 78, 35‐year‐old man, new HIV diagnosis)

#### 3.5.2 Anticipated skin‐disease stigma

Participants primarily expressed anticipated skin disease‐related stigma as it related to disclosure of their skin disease. Those who were able to cover and hide their skin disease did so, and those who could not, viewed the presence of skin lesions as automatic disclosure of their disease:

*Even going outside…. at the estate, I would put on socks and cover up with a leso [cloth], but even with sock, someone can still tell that your legs are swollen. It was embarrassing*. (Participant 76, 39‐year‐old woman, new HIV diagnosis)

#### 3.5.3 Experienced skin‐disease stigma

Many people with KS felt discriminated against because of their visible lesions, swellings or areas of discharge. Some participants were laughed at, asked to leave, abused or lost employment because of visible KS‐related skin changes:

*What I feared the most was how people were speaking to me, people didn't want me where they were because my leg was smelling, they would abuse me, that was my biggest fear*. (Participant 17, 32‐year‐old man, previous HIV diagnosis)

One participant specifically identified skin disease‐related stigma as the reason for losing employment. *“Where I had been working, my boss fired me because of those wounds – so these wounds were bleeding so much.”* (Participant 5, 34‐year‐old man, previous HIV diagnosis)

#### 3.5.4 Internalized skin‐disease stigma

Many individuals expressed embarrassment of the changes in their skin and drainage associated with KS, this internalized stigma led them to avoid contact with other people:

*I had some wounds which were discharging …I was stinking, the condition was ashaming me. Not that the friends were discriminating [against] me but I felt myself that it's not good to be where people are when you are not producing good smell*. (Participant 37, 40‐year‐old man, previous HIV diagnosis)

### 3.6 Intersectionality in KS stigma

Among people with KS, the experiences of HIV, cancer and skin disease stigma are intertwined in everyday experience, making it challenging to analyse these as isolated disease‐specific stigma experiences. The interviews reveal important manifestations of HIV‐related, skin disease‐related and cancer‐related stigma, and some participants described the complexities of their intersectional relationship. While many participants said HIV‐related stigma nullified other potential sources of stigma (e.g. cancer), others felt more stigmatized because they had HIV and cancer. Skin disease is stigmatizing in and of itself, and it is also a potentially identifiable visible manifestation of cancer and HIV. The experience of skin disease stigma thus shapes the experiences of HIV and cancer stigma. This is true for all three disease‐specific aspects of stigma, which become interwoven to create the experience of KS stigma:

*Since they have said it is just on the skin, my prayer is that I get well. Despite the fact that I have this other one [HIV] (laughs sarcastically), I hope to get well. This one [cancer] will disturb you! It does not please me, no (speaks in low tone)!* (Participant 82, 55‐year‐old woman, new HIV diagnosis)

*I was afraid. I thought now the cancer in combination with the HIV virus will take me very fast [lead to death] (laughs)*. (Participant 74, 29‐year‐old man, previous HIV diagnosis)

### 3.7 Role of stigma in KS diagnosis and treatment

Many participants experienced an initial loss of hope, fear of telling others and shame upon diagnosis that began to fade as the patient recovered or others became familiar with their condition. For some patients, stigma led to the delay of both diagnosis and treatment of KS.

When asked about avoiding the hospital because of staring and negative attention, *“That even prevented me from just going to where they were, because of flies and the discharge, I mean it was affecting me, and it prevented me from going to the hospital or anywhere else…even that was the reason I couldn't walk around.”* (Participant 17, 32‐year‐old man, previous HIV diagnosis)

In others, it was a motivating factor in seeking diagnosis and treatment to be cured more quickly.

*“…my friends had started being shy of intermingling with my friends because I was stinking, the condition was ashaming me. […] infact it motivated me to look for a solution for the problem so that I go on with my normal life.”* (Participant 37, 40‐year‐old man, previous HIV diagnosis)

### 3.8 Role of treatment in reducing stigma

When asked what he is expecting to change once he starts treatment, one patient says, “You know when I get better, I will be free…I will be free to interact with people, they can even call me for a job, or I can … do my work for my life to move forward.” (Participant 83, 32‐year‐old man, new HIV diagnosis)

One participant noted a significant change from initial diagnosis to time of illness improvement with treatment. Initially, no one cared for him, and many distanced themselves; however, when asked what people said when they saw him now, he responded *“I think they are just surprised to see me healed…they are just silent.”* (Participant 49, 34‐year‐old man, previous HIV diagnosis)

## 4 DISCUSSION

Our findings suggest that stigma is an important part of the lived experience of people with HIV‐associated KS in Kenya. The finding that overall stigma is highest around the time of KS diagnosis and declines longitudinally is supported by the quantitative and qualitative portions of our analysis. People with HIV‐associated KS have a unique experience because of the convergence of three co‐occurring potentially stigmatizing diseases (HIV, cancer and skin disease), yet their stories of stigma are dominated by HIV‐related stigma.

While longitudinal changes in stigma following cancer and skin disease diagnosis are not well studied, there is some prior work showing reductions of HIV stigma over time. A longitudinal study of the impact of stigma on quality of life among people living with HIV showed that HIV stigma was lower at 12 months than at baseline [27], though this was a secondary finding for which the reason was not fully explored. Here, we show that stigma among people with HIV‐associated KS declined following KS diagnosis during longitudinal evaluation and may reflect recovery with treatment. During semi‐structured interviews, people with HIV‐associated KS described experiences of stigma decline associated with chemotherapy initiation, resolution of their KS skin lesions and improvement of their overall health. Despite the observation that HIV is perceived by people with KS as the most stigmatizing aspect of their experience, skin disease may be an important driver of stigmatization in the community, since it is the most visible marker of cancer and/or HIV. In this way, it is possible that people with KS become less visible in the community as their skin lesions resolve and they perceive less overall stigma.

When examining the intersectionality of HIV‐related, cancer‐related and skin disease‐related stigma, people with KS identified HIV‐related stigma as a central barrier to KS diagnosis and treatment. The HIV‐related stigma impacted their lives more than both cancer‐related and skin disease‐related stigma. This is consistent with prior work in Kenya showing a higher proportion of women living with HIV reporting HIV stigma as compared to cervical cancer stigma [11]. Stories from this study corroborate this phenomenon, and participants articulated that stigma associated with their HIV diagnosis is stronger than their cancer or skin disease diagnosis. Interestingly, this relationship was not reflected in our quantitative evaluation, where the degree of HIV‐related, cancer‐related and skin disease‐related stigma was similar. While using the same measure to assess different components of stigma is a common approach to measuring intersectional stigma [28], it is possible that this approach was not sensitive enough to allow participants to distinguish between HIV‐related, cancer‐related and skin disease‐related stigma in the quantitative evaluation. This quantitative finding raises questions about whether this is the strongest instrument to quantitatively measure intersectional stigma in this setting, which warrants further investigation.

Among the stigma constructs from the Health Stigma and Discrimination Framework, anticipated HIV stigma related to HIV status disclosure was very common. This finding was consistent in the qualitative and quantitative analyses, where anticipated stigma was the highest sub‐score on the Berger‐aHSS scale for overall, cancer‐related, skin disease‐related, and HIV‐related stigma. Prior studies have similarly found anticipated stigma to be the highest sub‐score [29, 30, 31]. Anticipated stigma around HIV disclosure may be an important driver for stigma among people with HIV‐associated KS and a key barrier to the diagnosis and treatment of HIV‐associated KS. Anticipated HIV‐related stigma is also an important factor in the HIV care continuum and is associated with lower eagerness to begin ART [32]. For people with HIV‐associated malignancies, anticipated HIV‐related stigma may be a barrier to the diagnosis and treatment of both HIV and cancer.

We acknowledge that definitive conclusions about longitudinal changes in stigma over time are limited due to attrition bias, as only people with KS who survived are represented at later time points. However, the overall trend towards decreasing stigma persisted after accounting for within subject changes in stigma over time and adjusting for AIDS Clinical Trials Group (ACTG) stage at diagnosis and death at the end of the study period.

KS is often visible, presenting with overt clues identifying a person as having HIV and cancer that may lead to stigma. Although in this way KS is different from other HIV‐associated malignancies, the finding that HIV overshadows the intersectional nature of the stigma experience may be generalizable to other HIV‐associated malignancies. Similarly, people with other HIV‐associated malignancies may experience improvements in stigma following treatment and resolution of symptoms. One challenge to the generalizability is that stigma scores in this western Kenyan population were low relative to other populations. In our evaluation of stigma, the median overall stigma score was 28, indicating that most participants either strongly disagreed or disagreed with the statements about stigmatization in the Berger‐aHSS. Using quantitative evaluation of stigma alone, without the mixed‐methods approach used in this analysis, therefore, could potentially underestimate the burden of stigma in this context. Other studies quantitatively evaluating HIV stigma in the African context are mixed: some studies found universally high stigma for people living with HIV, while others found, similar to our study, low overall stigma [20, 33, 34]. Findings of overall low HIV stigma could be due to underreporting related to social desirability bias, cultural attitudes towards stigma in western Kenya or poor performance of adapted Berger‐aHSS scales in our context [34].

## 5 CONCLUSIONS

This mixed‐methods evaluation highlights the importance of stigma in the lived experience of people with HIV‐associated KS. While HIV‐related stigma may dominate individuals’ narratives about stigma, the intersectionality between skin, cancer and HIV still plays an important role in the experience of individuals living with a visible HIV‐related cancer. By evaluating KS stigma through a convergent mixed‐methods approach, our analysis underscores that experiences of stigma may be subtle, and quantitative evaluation of stigma may not adequately capture the experiences of intersectional stigma in certain contexts. Future research should focus on understanding whether stigma among people with HIV‐associated KS leads to differences in cancer care utilization and clinical outcomes.

## COMPETING INTERESTS

The authors have no competing interests to declare.

## AUTHORS’ CONTRIBUTIONS

EF, JM, IB, L Bogart and L Butler contributed to the conceptualization and design of the study. AS, HB, MLO, LC, TM, SK, EF and NB acquired the data. SC, RS, MG and DM analysed the data. SC, RS and EEF prepared the manuscript. EF, SC, RS, JM, L Bogart, L Butler, AS, HB, MLO, LC, TM, SK, NB, MG and DM reviewed and contributed to the final manuscript. All authors have read and approved the final version.

## Funding

Research reported in this publication was supported by the National Institute of Allergy and Infectious Diseases (NIAID), National Cancer Institute (NCI), and the Fogarty International Center in accordance with the regulatory requirements of the National Institutes of Health under Award Numbers, U54 CA190153, U54 CA25457, K23AI136579, K24AI141036, and D43TW009345‐09S7 awarded to the Northern Pacific Global Health Fellows Program. The funders did not have a role in design of the study, analysis, interpretation of data, or in writing the manuscript. The content is solely the responsibility of the authors and does not necessarily represent the official views of the National Institutes of Health.

## DISCLAIMER

The content is solely the responsibility of the authors and does not necessarily represent the official views of the National Institutes of Health.

## Supporting information


**Supplement A**: Quantitative methods and results, supplemental

**Supplement B**: Semi‐structured interview guides

## Data Availability

The data that support the findings of this study are available on request from the corresponding author.
