# Supplementary material for: Telling the story of intersectional stigma in HIV‐associated Kaposi's sarcoma in western Kenya: a convergent mixed‐methods approach
Source: J Int AIDS Soc. 2022 Jul 12;25(Suppl 1):e25918. doi: 10.1002/jia2.25918 (PMC9274375; doi:10.1002/jia2.25918)
Supplement: Supplementary file 1 — Supplement A: Quantitative methods and results, supplemental [file JIA2-25-e25918-s002.docx]

**Supplement A**

**Quantitative Methods, Supplemental**

**Data Collection Procedures**

Abridged, Berger HIV Stigma Scale:

As part of the adaptation process, we revised the Berger-aHSS to ask about 1) overall stigma, 2) cancer-related stigma, 3) skin disease-related stigma, by replacing “HIV” with “this illness”, “cancer,” and “skin condition” in all instructions and questions [1]. The term “this illness” was used to assess overall stigma at baseline, with the following, “These questions relate to your overall general health and illness, which can mean different things to different people. Please answer these questions considering what “illness” means to you - this may be one condition or many conditions.”

Sampling Structure and Censoring: Participants in the parent study were asked to complete the 4 adapted Berger-aHSS scales between February 2019 and December 2020. Because this stigma sub-study was initiated in February 2019, stigma measures were completed by participants starting at varying timepoints following KS diagnosis (ranging from 0 weeks to 128 weeks). Participants diagnosed with KS after February 2019 completed the stigma measures starting at the time of KS diagnosis (0 weeks). Follow-up study visits collecting the adapted Berger-aHSS scales occurred at 16-week intervals for the first 128 weeks (~2.5 years) after KS diagnosis.

Analysis: We assessed missingness in the data and compared participant characteristics among participants who completed a stigma measure and those who did not complete a stigma measure, including death at the end of the study period. We used linear regression to evaluate independent associations between total stigma score and age, gender, CD4 T+ cell count, and ACTG Stage at the time of KS diagnosis and death at the end of the study period. We then performed a linear mixed-effects model with a gaussian link, with random effects for the intercept for each participant, which was fully adjusted for all covariates.

A possible driver of attrition bias in this study is related to death prior to the end of the study period resulting in loss to follow-up. We used sensitivity analyses to assess whether the results of the analysis persisted in subgroups of participants who were alive at the end of the study period and who had died prior to the end of the study period (224 weeks). We performed linear mixed-effects models with a gaussian link, with random effects for the intercept for each participant, adjusted for age, gender, CD4 T+ cell count, and ACTG stage among these two subgroups (participants who were alive at the end of the study period and participants who had died prior to the end of the study period).

**Quantitative Results, Supplemental**

The proportion of eligible participants who completed the stigma quantitative surveys declined over time, 85.5% (53/62) completed the quantitative survey at the baseline visit, and 23.0% (29/126) completed the quantitative survey at the 128-week visit (Supplement A). The proportion of eligible participants who died prior to completing the Berger-aHSS scales also increased over the study period. At week 32, 6.9% (5/72) had died, whereas by week 128, 40.5% (51/126) had died.

In univariate analysis, age (β = -0.28, p= 0.01), gender (β = 4.68, p= 0.02), CD4 T+ cell count (β = -0.01, p= 0.04), and time from KS diagnosis (β = -0.09, p= <0.001) were all statistically significantly associated with total overall stigma score at the p=0.05 level. In the final adjusted multivariate model, accounting for participant as a random effect, only time from KS diagnosis (β = -0.15, p= <0.001), and gender were significantly associated with total overall stigma score (β = 6.54, p= 0.048). In the sensitivity analysis, we evaluated participants alive at the end of the study period (N=81) separately from participants who died prior to the end of the study period (N=13). Both groups had a decrease in stigma scores over time following KS diagnosis (β = -0.15, p= <0.001 for those still alive and β = -0.03, p= 0.8 for those who had died), but this was only statistically significant in the group of patients still alive at the end of the study.

Supplemental Table 1: Unadjusted and adjusted models of the association between time from KS diagnosis and overall sigma score among people with HIV-associated KS in Kenya.

|  | **Unadjusted** | | | **Adjusted Model** | | |  |
| --- | --- | --- | --- | --- | --- | --- | --- |
| **Characteristic** | **Beta** | **95% CI***^1^* | ***P***  **value** | **Beta** | **95% CI***^1^* | ***P***  **value** | |
| Age, years | -0.28 | -0.50, -0.07 | 0.011 | -0.21 | -0.54, 0.12 | 0.21 | |
| Gender |  |  |  |  |  |  | |
| Male | — | — |  | — | — |  | |
| Female | 4.68 | 0.75, 8.60 | 0.02 | 6.54 | 0.12, 12.95 | 0.048 | |
| CD4+ T-cells,  cells/μl at Diagnosis | -0.01 | -0.02, 0.00 | 0.043 | -0.0028 | -0.014, 0.0086 | 0.63 | |
| ACTG Stage |  |  |  |  |  |  | |
| T1 | — | — |  |  |  |  | |
| T0 | -0.71 | -6.2, 4.8 | 0.8 | -2.44 | -14.0, 9.11 | 0.68 | |
| Death at End of Study |  |  |  |  |  |  | |
| Dead | — | — |  |  |  |  | |
| Alive | -7.14 | -14, 0.03 | 0.052 | -7.41 | -17.3, 2.46 | 0.14 | |
| Time from KS Diagnosis, weeks | -0.09 | -0.13, -0.04 | <0.001 | -0.15 | -0.21, -0.098 | <0.001 | |

Legend: *^1^*CI = Confidence Interval

**References**

1. Jeyaseelan L, Kumar S, Mohanraj R, Rebekah G, Rao D, Manhart LE. Assessing HIV/AIDS stigma in south India: Validation and abridgement of the Berger HIV Stigma scale. AIDS Behav. 2013;17(1):434-43.
