# Supplementary material for: Telling the story of intersectional stigma in HIV‐associated Kaposi's sarcoma in western Kenya: a convergent mixed‐methods approach
Source: J Int AIDS Soc. 2022 Jul 12;25(Suppl 1):e25918. doi: 10.1002/jia2.25918 (PMC9274375; doi:10.1002/jia2.25918)
Supplement: Supplementary file 2 — Supplement B: Semi‐structured interview guides [file JIA2-25-e25918-s001.docx]

# Semi-structured Interview guide: ks patient diagnosis

## **INTRODUCTION**

Thank you for agreeing to talk with us today.  My name is [name] and I work at AMPATH/Moi Teaching and Referral Hospital. We are talking with people like yourself to gather thoughts and ideas about diagnosis for Kaposi’s sarcoma. Specifically, we want to hear your thoughts on how you were diagnosed, and factors that might have either prevented you from getting diagnosed sooner, or things that helped you get diagnosed when you did. We are talking with other patients, as well as medical providers, to help figure out how to best help patients.  We are talking with you, because you are an expert, and have lived through this process of being diagnosed with Kaposi’s sarcoma. Your experiences, thoughts, and opinions will help us to think about how we may be able to help patients with Kaposi’s sarcoma in the future.

There are no right or wrong answers. I am most interested in your experience and perspective, which is what I would like to learn about. I am sorry if I cut you off at any point – there’s a lot I would like to ask you, and I would like to make sure we cover a little bit of everything.

All information that you share with me today is completely confidential and will only be used to help us with our research. What we discuss here will not be talked about with your healthcare providers. Once we complete our study, we will destroy the link between your name and your answers to our interview questions.  With your permission, I will record this interview to supplement my notes, but the recording will be erased after we complete the study. To protect your confidentiality, no one outside of the research team will see the notes or listen to the recording.  We will not provide any information that will allow linking of your name to your specific comments.

The interview is completely voluntary, you can stop participating at any time, and you can skip any questions you’d rather not answer.  The interview should take approximately 60 minutes.

I have brought a drink and some biscuits for you, which you can feel free to take at any time. The washroom is just down the hall. If you need to stop at any time, please let me know. Do you have any questions about the study at this time?

To give your consent to participate in the interview, we would need you to read over this form with us and sign and date it. [PROVIDE CONSENT FORM TO VOLUNTEER AND READ ALOUD; ANSWER ANY QUESTIONS BEFORE OBTAINING CONSENT]

Shall we begin? I’m going to turn the voice recorder on now.

Today is ____ (date), the interviewer is _____, and the participant RCA ID is _________. The interview is taking place at _____ (site), we are beginning the interview at _______ [time].

## **TOPIC 1: KS Diagnosis Information**

**General Knowledge about KS**

1. To get started, can you tell me about the condition that you had on your skin [or mouth]?

Possible probes:

- What do you think this condition is?
- What do you understand about your condition?
- Has any provider explained what this condition is?
- What have they told you?

**Knowledge of Symptoms of KS**

2. What symptoms of this condition did you first notice?

3. Did you yourself notice the spots or did someone else notice them? If someone else, who was it?

4. When did you start noticing your symptoms? How many months or years?

5. What did you do when you first noticed these symptoms?

**Knowledge of how to receive care for KS**

6. Please describe the people who you either sought advice from or who gave you advice about your condition. Who were they? What were their recommendations/advice? What did you do with that advice?

Possible probes:

- What did those people say about your condition?
  - Family members
  - Friends
  - Community members
  - Traditional healer
  - Health worker (ie. Doctor, Nurse)

**Relationship between HIV and KS**

7. Did you know your HIV status before you were diagnosed with this condition/KS? Tell me how your knowledge of HIV changed your concern for these lesions, if at all?

## **TOPIC 2: KS Diagnosis Motivation**

**Beliefs about KS**

8. When you noticed the spots, what did you believe was causing this condition?

9. How common do you think this condition is in your community? Who gets this condition?

10. What are some of the beliefs that people in your community have about this condition?

**Worries and fears around KS**

11. Did you feel you needed to worry about the spots? *Why? Why Not?*

12. Did you ever think these spots might be cancer? If so, did this prevent you from wanting to get a diagnosis?

13. Once you noticed the spots on your skin, are there any fears you had about getting a positive diagnosis?

14. Once you got your positive result for this condition /Kaposi’s sarcoma, what was your biggest fear in that moment?

**Expectations about KS**

15. What would happen if you did not get diagnosed with this condition?

16. How has this diagnosis changed your life?

**Barriers to KS diagnosis**

17. Sometimes people may have concerns or difficulties in getting diagnosed with this condition. What are challenges you had in getting diagnosed?

18. Say that [ANSWER TO 17] was not an issue. What else would still stand in your way of receiving a diagnosis?

Possible Probes:

- Once the patient has finished responding, **ASK** the patient specifically about the following barriers
  - Lack of family / friend’s support
  - Worries about cost
  - Not feeling well enough to attend clinic
  - Feeling hopeless about condition
  - Fear of disclosing HIV status
  - Negative interactions with providers
  - Transportation issues attending clinic
  - Long wait times at clinic
  - Biopsy service not available at clinic
  - Long delay to receive pathology results

**Facilitators for KS diagnosis**

19. What are somethings that have helped you get diagnosed with this condition?

Possible Probes

- Once patient has finished responding, **ASK** about specific facilitators
  - Has financial support been helpful for you?
  - Have family, friends or others in your community been helpful in getting a diagnosis?
  - Seeing or knowing other people with this condition?
  - Public health campaigns about this condition? (ie. Radio, TV, poster, newspaper)
  - Your motivation to understand what is causing it?

## **Topic 3: KS Diagnosis Behavior and skills**

**Logistical challenges in reaching clinic**

20. How confident are you in your ability to go to the clinic with a health concern that you have?

21. Describe the feelings you have about coming into clinic?

Possible probes:

- What things about coming into clinic worried you?
- What things prevented you from coming into the clinic?

**Self-Efficacy**

22. How confident are you in handling unexpected events?

For example, how well were you able to handle receiving this positive diagnosis?

23. When faced with a difficult situation, how well do you come up with solutions?

For example, after this diagnosis, were you able to come up with plans for how to handle this new diagnosis and treatment?

## **Topic 4: Intervention**

We are exploring different ways of supporting patients to make it easier to get diagnosed with this condition/ KS. We’d like your opinion on a few things.

- If you were to design any intervention to make it easier for people to be diagnosed with this condition, what would it be?
- Would public messages about this condition (such as newsprint, radio, television campaigns) help someone to be diagnosed earlier? Which kind of campaign is best? (radio vs TV vs other)
- Before you noticed the spots, did a provider/doctor fully undress you to do a physical exam? After you noticed the spots, when you came into clinic with these lesions, did providers complete a full physical exam?
- What kind of knowledge about KS would have helped you notice the spots sooner? What kind of knowledge would have helped you come in sooner once you saw the spots?
- What kind of role could community health workers have in helping KS be diagnosed earlier?

## **Topic 5: Wrap-Up**

- Is there anything else related to your diagnosis for KS that I didn’t ask you that you would like to talk about?
- The interview has ended. The time is _____

# semi-structured Interview guide: ks patient chemotherapy

## **INTRODUCTION**

Thank you for agreeing to talk with us today. My name is [name] and I work with AMPATH/Moi Teaching and Referral Hospital. We are talking with people like yourself to share with us thoughts and ideas about Kaposi’s sarcoma treatment. Specifically, we want your opinion on how to best understand how to deliver treatment for Kaposi’s Sarcoma. We are talking with other patients like yourself that have been treated for Kaposi’s sarcoma, as well as medical providers to help figure out how to best address this issue. We are talking with you, because you are an expert, the type of person whom future program will target. Your experiences, thoughts, and opinions will help us to design a successful program.

All information that you share with me today is completely confidential and will only be used to help us with our research. What we discuss here will not be talked about with your healthcare providers. Once we complete our study, we will destroy the link between your name and your answers to our interview questions. With your permission, I will audio record this interview to supplement my notes, but the recording will be erased after we complete the study. To protect your confidentiality, no one outside of the research team will see the notes or listen to the recording. We will not provide any information that will allow linking of your name to your specific comments.

The interview is completely voluntary, you can stop participating at any time, and you can skip any questions you’d rather not answer. We will have some time at the end to go over any questions you might have.

I have brought a drink and some biscuits for you, which you can feel free to take at any time. The washroom is just down the hall. If you need to stop at any time, please let me know. Do you have any questions about the study at this time?

To give your consent to participate in the interview, we would need you to read over this form with us and sign and date it. [PROVIDE CONSENT FORM TO VOLUNTEER AND READ ALOUD; ANSWER ANY QUESTIONS BEFORE OBTAINING CONSENT]

Shall we begin? I’m going to turn the voice recorder on now.

Today is ____ (date), the interviewer is _____, and the participant RCA ID is _____. The interview is taking place at _____ (site), we are beginning the interview at _______ [time].

## **TOPIC 1: ks TREATMENT INFORMATION**

***General knowledge about KS***

1. To get started, can you tell me about the condition that you had on your skin [or mouth]? What do you think the condition is?

Possible probes:

- What do you understand about the spots on your skin / your condition?
- Has any provider explained what this condition is? What have they told you?

2. What do you think the cause of the condition is?

***Knowledge about KS treatment***

3. What do you understand about treatment for this condition?

4. What medicines, if any, do you need to take for this condition?

5. Some people might use chemotherapy to treat this condition. Have you heard of chemotherapy?

Possible probes:

- What do you know about chemotherapy?
- Have you heard of any chemotherapy, like BV or Doxil?

***Knowledge of chemotherapy logistics and prognosis***

6. What is the process of receiving treatment?

Possible probes:

- How often you need to take the treatment?
- Do you still have to take your ARVs while you are receiving treatment?

7. What would happen if you did not treat this condition?

8. Where should someone who has this condition go to get treatment?

Possible probes:

- Is there a particular clinic or doctor someone should go to?
- What are your thoughts on seeking care from a traditional healer? Do you think they can help, and why or why not?

## **TOPIC 2: KS Treatment motivation**

***Barriers to chemotherapy initiation***

9. Sometimes people may have difficulties regarding starting chemotherapy. What difficulties did you have?

10. I’ve heard some other patients talking about challenges starting chemotherapy. Other than the difficulties you just mentioned, what other barriers or challenges did you experience?

Possible probes:

- Once the patient has finished responding, **ASK** the patient specifically about the following barriers
  - Time required, such as time away from home or work
  - Fear of disclosing HIV status by seeking treatment
  - Stigma around having cancer / receiving chemotherapy
  - Concern about family or friends’ reaction
  - Concern about cost of transport
  - Concern about treatment cost
  - Concern about NHIF covering costs
  - Concerns about the chemotherapy itself, such as side effects
  - Not feeling healthy enough to go to clinic
  - Feeling hopeless about treatment
  - Long wait times at the clinic
  - Negative interactions with staff at the clinic

***Facilitators to chemotherapy initiation***

11. Tell me what has been able to help you start, or would be helpful for you to start?

Possible probes:

- Has financial support been helpful?
- Have friends, family, or other community members been helpful?
- Were there any public health campaigns around cancer or chemotherapy that were helpful?
- Your personal feeling that chemotherapy will be helpful?

***Barriers to chemotherapy adherence (SKIP IF PATIENT HAS NOT STARTED CHEMO*)**

12. Sometimes people may have or difficulties continuing chemotherapy once they have started. What are challenges you had in continuing chemotherapy? [Allow for open-ended response]

13. I’ve heard some patients talking of other challenges in continuing chemotherapy once you’ve started. Other than the difficulties you just mentioned, what other difficulties have you had in staying on chemotherapy?

Possible Probes:

- Once the patient has finished responding, **ASK** the patient specifically about the following barriers to adherence:
  - Time required, such as time away from home or work
  - Fear of disclosing HIV status by seeking treatment
  - Stigma around having cancer / receiving chemotherapy
  - Concern about family or friends’ reaction
  - Concern about cost of transport
  - Concern about treatment cost
  - Concern about NHIF: enrollment, covering costs, keeping active
  - Concerns about the chemotherapy itself, such as side effects
  - Not feeling healthy enough to go to clinic
  - Feeling hopeless about treatment
  - Long wait times at the clinic
  - Negative interactions with staff at the clinic

***Facilitators to chemotherapy adherence (SKIP IF PATIENT HAS NOT STARTED CHEMO*)**

14. What are somethings that have helped you continue chemotherapy?

Possible probes:

- Has financial support been helpful?
- Have friends, family, or other community members been helpful?
- Were there any public health campaigns around cancer or chemotherapy that were helpful?
- Your personal feeling that chemotherapy will be helpful?

***Hopes and Fears of Chemotherapy***

15. What would you/did you hope to achieve by taking chemotherapy? If that outcome happened, how would it change your life?

Possible probes:

- *Taking chemotherapy would allow me to…*
  - Work again?
  - Take care of my family?
  - Reduce pain?
  - Reduce skin lesions?
  - Prolong my life?

16. What is your biggest fear in taking chemotherapy?

Possible probes:

- Have you seen or heard of bad effects of chemotherapy?
- Have you experienced any side effects of taking chemotherapy?

***Social, financial, and medical support for KS treatment***

17. Are there people who are supportive of your care for KS?

Possible probe:

- Is there anyone who helps attend chemotherapy appointments, such as family, friends, or other community members?

18. How are you paying for your chemotherapy? How much does it cost? How has that affected your chemotherapy treatment?

Possible probe:

- Do you have NHIF or pay out of pocket
- If you have NHIF, how easy/hard was it to get NHIF? Has that been helpful in receiving chemotherapy?

19. Can you describe a positive experience and a negative experience you remember with a healthcare worker providing chemotherapy?

Possible probe:

- How did those interactions affect your care?
- Did you ever feel discriminated against by a health worker?

## **Topic 3: KS Treatment behavior and skills**

***Logistics of attending oncology visits***

20. Can you tell me about a time that you might have missed an oncology visit or a chemotherapy infusion? Why?

21. Are there certain skills or strategies you’ve developed that help you make it to your care visits?

Possible probes:

- When you kept oncology visits, how did you achieve this?
- Did you have appointment reminders?
- Did any friends or family help you remember appointments?
- How did you arrange transportation?
- Are there other things you think could help you make it to appointments?

***Self-Efficacy***

22. If something were to get in the way of you making it to an appointment or receiving chemotherapy, how confident are you in your ability to come up with a solution?

23. How confident are you in keeping track of your chemotherapy appointments?

## **Topic 4: Intervention**

We are exploring different ways of supporting patients on chemotherapy. We’d like your opinion on a few things.

- Do you have any ideas for what would help patients start or continue with chemotherapy?
- We are thinking about creating a support group for people with this condition to meet and talk with each other. Is that the sort of thing you would be interested in participating in? Would you prefer it occur inside of outside of the clinic, and why? Run by a nurse or counselor, and why? Weekdays or weekends, and why?
- Would it be helpful for someone to remind you about upcoming appointments? Would you prefer to have a phone call or text, and why?
- Would you think it would be helpful to have a peer navigator to help you on the days you come to clinic for oncology?
- Would you be interested in having a counselling session regarding chemotherapy? What should the counseling sessions cover? (Example topics: what questions I should be asking the provider, how to navigate NHIF, what to expect from the treatment, side effects)
- Would you prefer a 1:1 counselling session regarding chemotherapy, or a group counseling session regarding chemotherapy? Why?

## **Topic 5: wrap-up**

Tell The interview has ended. The time is _____
